# Management of *Clostridioides difficile* infection: an Italian Delphi consensus

**DOI:** 10.1093/jac/dkae179

**Published:** 2024-07-13

**Authors:** Matteo Bassetti, Antonio Cascio, Francesco Giuseppe De Rosa, Marianna Meschiari, Roberto Parrella, Nicola Petrosillo, Alessandro Armuzzi, Flavio Caprioli, Francesco Dentali, Marcello Pani, Alberto Pilotto, Umberto Restelli, Maurizio Sanguinetti

**Affiliations:** Infectious Diseases Unit, IRCCS Ospedale Policlinico San Martino, 16132 Genoa, Italy; Department PROMISE—Infectious and Tropical Diseases Unit, AOU Policlinico “P. Giaccone”, University of Palermo, 90127 Palermo, Italy; Department of Medical Sciences, University of Turin, 10126 Turin, Italy; Department of Infectious Diseases, Azienda Ospedaliero-Universitaria di Modena, Policlinico di Modena, University of Modena and Reggio Emilia, 41125 Modena, Italy; Unit of Respiratory Infectious Diseases, Cotugno Hospital, Azienda Ospedaliera dei Colli, 80131 Naples, Italy; Infection Prevention and Control Service, Fondazione Policlinico Universitario Campus Bio-Medico, 00127 Rome, Italy; IBD Unit, IRCCS Humanitas Research Hospital, Via A. Manzoni 56, Rozzano, 20089 Milan, Italy; Department of Biomedical Sciences, Humanitas University, Via Rita Levi Montalcini 4, Pieve Emanuele, 20072 Milan, Italy; Department of Pathophysiology and Transplantation, Università degli Studi di Milano, 20133 Milan, Italy; Gastroenterology and Endoscopy Unit, Fondazione IRCCS Cà Granda, Ospedale Maggiore Policlinico di Milano, 20122 Milan, Italy; Division of Internal Medicine, Medical Center, Ospedale di Circolo & Fondazione Macchi, ASST Sette Laghi, 21100 Varese, Italy; Department of Medicine and Surgery, Insubria University, 21100 Varese, Italy; Hospital Pharmacy, Policlinico Universitario A. Gemelli IRCCS, 00168 Rome, Italy; Department of Interdisciplinary Medicine, ‘Aldo Moro’ University of Bari, 70121 Bari, Italy; Geriatrics Unit, Department of Geriatric Care, Neurology and Rehabilitation, Galliera Hospitals, 16128 Genova, Italy; LIUC—Carlo Cattaneo University, 21053 Castellanza, VA, Italy; Department of Laboratory and Infectious Sciences, Fondazione Policlinico Universitario A. Gemelli IRCCS, Largo A. Gemelli 8, 00168 Rome, Italy

## Abstract

**Background:**

*Clostridioides difficile* infection (CDI), a leading cause of nosocomial deaths, is a microbiota-mediated disease. As such, the use of broader spectrum antibiotics, such as vancomycin and metronidazole, can prime the gastrointestinal tract to become more prone to CDI recurrences. Fidaxomicin, a narrow-spectrum antibiotic, has been demonstrated to be superior in preventing recurrence and in preserving the intestinal microbiota; however, widespread employment worldwide has been hindered due to high acquisition costs.

**Objectives:**

To integrate the currently available guidelines on the management of CDI and to shed light on the timeliest employment of fidaxomicin.

**Methods:**

An expert panel was gathered to obtain consensus using Delphi methodology on a series of statements regarding the management of CDI and on appropriate antibiotic use.

**Results:**

Consensus was reached on 21 of the 25 statements addressing the management of CDI.

**Conclusions:**

Delphi methodology was used to achieve consensus on the management of CDI, on the identification of patients at risk of recurrences or severe infection, and on the most appropriate use of fidaxomicin, with the final aim of fostering clinical practice application of treatment algorithms proposed by previous guidelines, in absolute synergy. It could be an important tool to promote more appropriate and cost-effective CDI treatments in European settings with limited resources, like Italy.

## Introduction

*Clostridioides difficile* infections (CDIs) are a leading cause of nosocomial deaths. This Gram-positive, spore-forming and toxin-producing intestinal bacterium that infects the human gut potentially causing lethal diarrhoea has been designated an ‘urgent threat’ by the US CDC^1–3^ and it is under surveillance at European and Italian levels.^4,5^ The burden of these infections in Italy is underscored by the median hospital incidence density of healthcare-associated CDI (2.9 cases per 10 000 patient-days), which is more pronounced in tertiary care hospitals (5.8 cases/10 000 patient-days). This is further emphasized by the recurrence rate at 4 weeks (21% of patients) and the disparity in length of stay [16 (IQR = 13) versus 8 (IQR = 8) days; *P* < 0.001] between patients with CDI and those without.^6^

The alarming increase in infections caused by highly pathogenic variants of *C. difficile* runs parallel to the use of broad-spectrum antibacterial drugs. CDI is, indeed, a microbiota-mediated disease: disruptions in the gut microbiota are critical to CDI development, whereas the restoration of homeostatic bacterial diversity and abundance is essential for recovery.^6^ Dysbiosis, like the one triggered by broad-spectrum antibiotic uptake, is a precursor to infection, and its persistence often leads to disease recurrence.^7^ Broader spectrum antibiotics such as vancomycin and metronidazole are used to treat CDIs, but these antibacterial agents decimate the normal gut microbiome, paradoxically priming the gastrointestinal tract to become more prone to CDI recurrences^8–10^ Not surprisingly, treatment of CDI with either metronidazole or vancomycin is associated with recurrence in 20%–30% of patients, which then provides a 50%–60% likelihood of further recurrence.^11^

In 2011, the macrocyclic antibiotic fidaxomicin became available to treat CDIs.^12^ Fidaxomicin selectively targets *C. difficile* without effectively killing crucial gut commensals such as Bacteroidetes, which crowd the human gut microbiome providing protection against *C. difficile* colonization.^13^ Fidaxomicin is a narrow-spectrum, macrocyclic antibacterial agent with minimal systemic absorption. It showed higher *in vitro* activity against *C. difficile* than vancomycin, with a more prolonged post-antibiotic effect and reduced sporulation and toxin production *in vitro* and *in viv*o.^14^ Most importantly, two prospective randomized controlled trials demonstrated non-inferiority of fidaxomicin versus vancomycin for clinical cure of CDI.^11,12^ Although fidaxomicin can be associated with treatment failure and relapse after primary infection, it has been demonstrated to be superior in preventing recurrence and in preserving the intestinal microbiota thanks to its narrow-spectrum activity.^15^ Of note, fidaxomicin-treated hospital inpatients proved to be less likely to contaminate their environment (36.8%) than patients treated with metronidazole and/or vancomycin (57.6%).^16^

Despite key advances in therapeutic strategies, CDI remains challenging for clinicians worldwide:^15^ beside the management of infrequent cases of fulminant colitis, which carry a high risk of mortality, the most difficult task consists in preventing recurrent infections. Heterogeneity in definitions used for severe and potentially recurrent CDI (rCDI) has been a confounding factor when assessing treatment guideline recommendations and trial outcomes. Consensus between the IDSA, the Society for Healthcare Epidemiology of America (SHEA) and the ESCMID regarding optimal treatment of initial and first recurrence of non-severe CDI has only recently been reached.^15^ The recent IDSA/SHEA update suggests fidaxomicin preferentially over vancomycin for initial CDI,^17^ and the latest ESCMID guidelines^18^ concur with this recommendation, with vancomycin being acceptable for a first episode, but metronidazole only if the other agents are unavailable.

Although fidaxomicin performed favourably against vancomycin in clinical trials of CDI and has been suggested preferentially over broader spectrum antibiotics by the most recent guidelines, widespread use worldwide has been hindered due to its higher cost. Various recent studies, either industry-supported^19–23^ or not,^24,25^ showed in different settings and using different health economic models that CDI treatment with fidaxomicin can reduce global healthcare costs.

Extended-pulsed fidaxomicin therapy was more cost-effective than vancomycin for first-line treatment of CDI in older patients.^20^ In fact, higher drug acquisition costs for fidaxomicin were found to be compensated by lower hospitalization costs driven by fewer recurrences, lower costs of complications, and fewer GP visits versus vancomycin. In Italy, for instance, a real-world study showed that the mean cost of a recurrent episode of CDI amounts to €9504.87 ± €8614.11.^26^ To mitigate the higher acquisition costs of fidaxomicin compared with those of vancomycin, the ESCMID 2021 update on the treatment guidance for CDI suggests applying a risk-stratification strategy in case of economic restraints.^18^ Identifying patients at risk, however, is challenging. Several prediction models have been developed, yet none has been widely adopted in clinical practice.

This study aimed to integrate current guidelines on the treatment of CDIs focusing on practical issues where clinical evidence is still limited. A panel of Italian experts using the Delphi methods approach, was gathered to obtain consensus on a series of statements addressing (i) the proper use of fidaxomicin; (ii) the impediments to the practical implementation in Italy of the evidence coming from the existing CDI guidelines, and (iii) the strategy of prevention and treatment of recurrent infections. In order to obtain consensus, the Delphi method was employed; this widely accepted technique, built on evidence-based medicine, adopts consecutive iterations using a survey until consensus is reached,^27^ allowing clinical recommendations for those areas, like *Clostridioides*-mediated infections, where clinical-based evidence is still insufficient.

## Methods

### Study design

**Figure 1.**
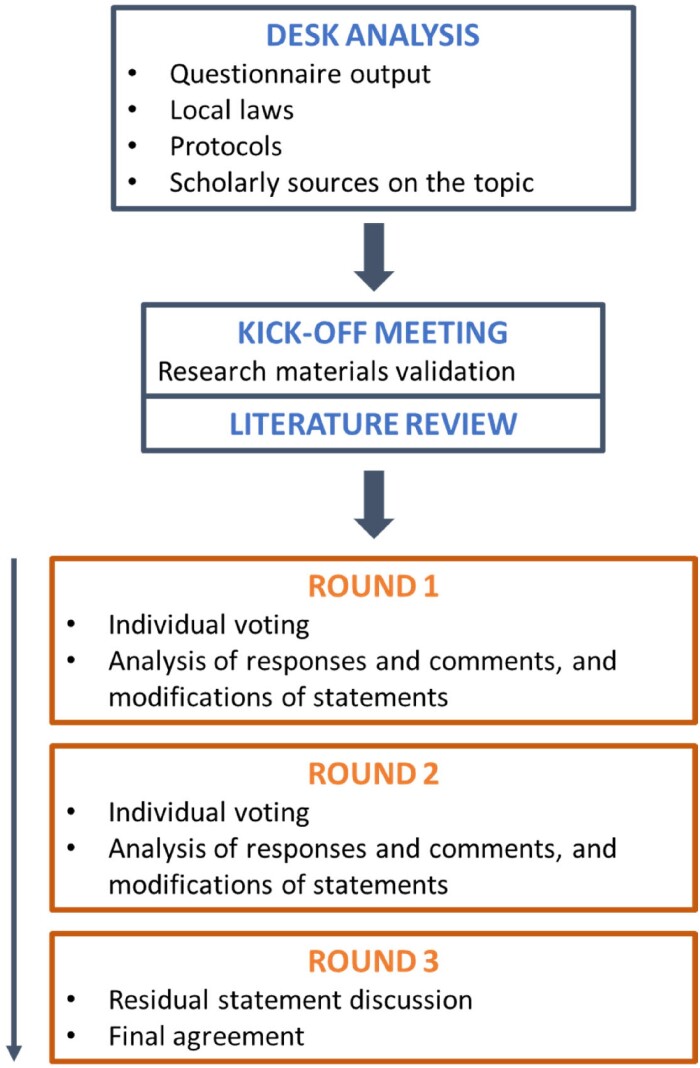
Overall flow of the Delphi process that was employed in this study. This figure appears in colour in the online version of *JAC* and in black and white in the print version of *JAC*.

A modified Delphi process (Figure 1) was organized in the following phases, which were run over a period of 10 months (from June 2023 to March 2024):

1. *Desk analysis:* a preliminary list of principles of starting points and research questions was drafted by research assistants following analysis of scholarly sources on the topic, local Italian laws addressing the management of CDIs, and the output of a questionnaire covering clinical and organizational subjects submitted to healthcare professionals dealing with CDIs in their daily practice and distributed in the nine main Italian regions: 34 clinicians (including 6 gastroenterologists, 1 geriatrician, 22 infection disease specialists; 5 internists), 4 microbiologists, 13 hospital pharmacists and 1 chief medical officer.
2. *Identification and selection of a panel of experts:* 13 Italian experts (the Scientific Board) were identified by their experience in treatment of CDIs, relevant publications, academic status, clinical practice at recognized centres of excellence, experience in clinical trials, and participation in national and international conferences. The experts were representative of the national territory (seven from Northern Italy, three from Central Italy, and three from Southern Italy); they were specialized in gastroenterology (*n* = 2), infection diseases (*n* = 6), internal medicine (*n* = 1), microbiology (*n* = 1), health economics and healthcare management (*n* = 1), geriatrics (*n* = 1) and hospital pharmacy (*n* = 1).
3. *Kick-off meeting and literature review:* during the first meeting of the scientific board, the outputs of the desk analysis, including a preliminary list of draft statements, were presented, critically discussed and revised. A literature review of articles published in peer-reviewed journals allowed identification of red flags for referral on the treatment of CDIs. The search was performed online using the PubMed database from 2015 to 2023. Predefined keywords and inclusion criteria were used; these included: ‘fidaxomicin’, ‘recurrence’, ‘recurrent clostridium/clostridioides difficile’, ‘infection’, ‘vancomycin’ and ‘guideline’. Only studies published in the English language (unless a specific article in another language was considered relevant by the Board) were included. Letters and abstracts were excluded.
  After the kick-off meeting, 23 statements were drafted and submitted for the first round of online voting.
4. *First, second and third rounds of online Delphi voting:* In Delphi Round 1, the 13 experts were asked to express their judgement on the initial 23 statements. Voting was undertaken by email using a 5-point Likert scale to indicate the level of agreement on each statement: 1 = absolutely disagree, 2 = disagree, 3 = neither agree nor disagree, 4 = agree, 5 = totally agree. The collected answers were expressed as a percentage response for each item. A total cumulative agreement was defined as the sum of response percentages in items 4 (‘agree’) and 5 (‘absolutely agree’). For the purpose of this consensus, a total cumulative agreement ≥75% was considered a priori to represent consensus for each statement. This definition of agreement was based on standards used in previous Delphi studies^28–30^ During Round 1 voting, experts were also given the opportunity to provide comments and suggest additional items that may not have been included when developing the initial list of statements, with the aim of clarifying any redundancies or issues regarding comprehension or syntax of each statement.

Once Round 1 voting was completed, an analysis of responses and comments was performed. Statements that reached a total cumulative agreement >75% without receiving specific comments or requests of amendment/integration were considered finalized. Statements that reached a total cumulative agreement >75% and received specific comments or requests of rewording/rephrasing were modified accordingly before being submitted to the panel for Round 2 voting. Among statements that did not reach a consensus after Round 1, those that scored ≤75% without specific indications for amendment were excluded. Those that reached a total cumulative agreement >50% and specific indications were modified and/or integrated according to the feedback received before being submitted to the panel for Round 2 voting.

Round 2 voting followed the same process of Round 1. Except for those statements that were already finalized or excluded, each member of the panel expressed their level of agreement for each item and, if deemed necessary, provided comments.

In Round 3, residual statements were discussed in order to nail down a final wording for those statements that were agreed in Round 1 and Round 2 but were suggested to be amended or integrated. Statements that did not reach a consensus in Round 2 but reached a total cumulative agreement >65%, were voted again in Round 3.

An additional statement was added and voted during the revision phase of the manuscript in agreement with the editor’s recommendations.

## Results

The 23 statements that were drafted after the kick-off meeting (Table 1) spanned the following areas:

- diagnosis, including definition of severe infection, frail patient, and patients at risk of recurrences;
- management of CDIs in patients at high risk;
- benefits of fidaxomicin therapy compared with treatment with broader spectrum antibiotics;
- management of CDI and cost monitoring.

**Table 1.** Statements and results of voting

#	Round 1 statements	Results	Round 2 statements	Results	Round 3 statements	Results	TCA
1	CDI diagnosis Diagnosis of CDI should be made according to the recommended algorithms for CDI testing described in ESCMID diagnostic guidance document^31^	Agreement reached—final statement					100%
2	Patient management before and after confirmation of CDI Isolation measures for patients with (or suspected to have) CDI should be implemented in a timely way in agreement with ESCMID prevention guidance document^32^ and with 2017 IDSA/SHEA guidelines^33^	Agreement reached—final statement					100%
3	The prompt consideration of severe infection should be undertaken in conjunction with its principal risk factors: older age (>65 y old)^17,18,34^ hypoalbuminaemia prior to infection <2 g/dL^34^ presence of multiple comorbidities: IBD, chronic kidney failure, liver failure, obesity, diabetes, cardiovascular/pulmonary disease, parenteral nutrition, febrile neutropenia^34^	Agreement reached—statement to be amended	The prompt consideration of severe infection should be undertaken in conjunction with its principal risk factors: older age (>65 y old)^17,18,34^ hypoalbuminaemia prior to infection <2.5 g/dL^1,34–37^ presence of comorbidities or conditions: IBD, chronic kidney failure, liver failure, diabetes, cardiovascular/pulmonary disease^34^ Zar score ≥2^38,39^	Agreement reached—final statement			100%
4	Identification of patients at risk of recurrences holds significant importance in establishing the therapeutic approach. The foremost risk factors for rCDI are: Severe form of infection^17^ Immunocompromised patients: transplanted, on oncological/oncohaematological treatments, on immunosuppressive therapies, HIV-positive/AIDS, other immunodeficiencies^17,18^ Older age (>65 y old)^17,18,34^ IBD, chronic kidney failure, liver failure, obesity, diabetes, cardiovascular/pulmonary disease, parenteral nutrition^34^ Healthcare-associated CDI^18,34^ Prior hospitalization in the last 3 mo^34^ Recent exposure to fluoroquinolones, cephalosporins, carbapenems, clindamycin^18^ Recent use of PPIs^18,34^ Use of concomitant antibiotics started during/after CDI diagnosis^18,34^ and (a) prior CDI episode(s)^18,34^	Agreement reached—statement to be amended	Identification of patients at risk of recurrences holds significant importance in establishing the therapeutic approach. The foremost risk factors for rCDI with strong evidence are: Older age (>65 y old)^17,18,34^ IBD^34,40^ Immunocompromised patients: transplanted,^14^ on oncological/oncohaematological treatments,^41^ on immunosuppressive therapies,^42^ HIV-positive/AIDS, other immunodeficiencies^17,18^ Healthcare-associated CDI^18,34^ Prior hospitalization in the last 3 mo^34^ Recent use of PPIs^18,34^ and (a) prior CDI episode(s)^18,34^ Other established risk factors are: Severe form of infection^17^ Chronic kidney failure, liver failure, diabetes, cardiovascular/pulmonary disease, parenteral nutrition^18^ Recent exposure to fluoroquinolones, cephalosporins, carbapenems, clindamycin^18^ And use of concomitant antibiotics started during/after CDI diagnosis^18,34^	Agreement reached—statement to be amended.	Identification of patients at risk of recurrences holds significant importance in establishing the therapeutic approach. The foremost risk factors for rCDI with strong evidence are: Older age (>65 y old)^17,18,34^ IBD^34,40^ Immunocompromised patients: transplanted,^14^ on oncological/oncohaematological treatments,^41^ on immunosuppressive therapies,^42^ HIV-positive/AIDS, other immunodeficiencies^17,18^ Healthcare-associated CDI^18,34^ Prior hospitalization in the last 3 mo^34^ Recent use of PPIs^18,34^ Recent exposure to fluoroquinolones, cephalosporins, carbapenems, clindamycin^9,18^ and (a) prior CDI episode(s)^18,34^ Other established risk factors are: Severe form of infection^17^ Chronic kidney failure, liver failure, diabetes, cardiovascular/pulmonary disease, parenteral nutrition^34^ And use of concomitant antibiotics started during/after CDI diagnosis^18,34^	Agreement reached—final statement	100%
5	In IBD patients, recurrences are more likely in correspondence with: recent antibiotic therapy, 5-aminosalicylic acid or steroid use, and biologic therapy, in particular with infliximab^35^	Agreement reached—statement to be amended.	In IBD patients, it must be considered that: Since CDI is the most important cause of an IBD flare, all IBD patients with worsening of underlying diarrhoea or symptoms of colitis, should be tested for CD^43^ Recurrences are more likely in correspondence with: recent antibiotic therapy, steroid use, infliximab and adalimumab.^40,43^ Evidence is conflicting on other immunosuppressive drugs^40^	Agreement reached—final statement			100%
6	Frailty condition should be taken into proper account in the management of CDI, because of higher risks of negative outcomes reported in frail patients^1^	Agreement reached—statement to be amended	Frailty condition should be taken into proper account in the management of CDI, because of higher risks of negative outcomes reported in frail patients.^44^ In persons >65 y with CDI, multidimensional frailty level predicts mortality (at 90 d) more accurately than chronological age and disease severity^44^	Agreement reached—final statement			100%
Ex7	In persons >65 y with CDI, multidimensional frailty level predicts mortality (at 90 d) more accurately than chronological age and disease severity^1^	Agreement reached—statement to be amended		Incorporated into statement 6			100%
7 (Ex 8)	In hospitalized older patients with CDI, multidimensional frailty should be accurately assessed through validated and calibrated Comprehensive Geriatric Assessment (CGA)-based frailty tools such as the Multidimensional Prognostic Index (MPI)^1,36,37^ or its screening short version BRIEF-MPI^38^	Agreement reached—statement to be amended	In hospitalized older patients with CDI, multidimensional frailty should be accurately assessed through validated frailty tools such as the Multidimensional Prognostic Index (MPI)^44–46^ or its screening short version BRIEF-MPI^47^	Agreement reached—final statement			100%
8			In patients at first CDI episode with high risk of recurrence, fidaxomicin is recommended, since it is associated with a higher cure and significantly sustained response^11,12^	83%	In patients at first CDI episode with high risk of recurrence, fidaxomicin is recommended, since it is associated with significantly higher sustained response^11,12^		100%
9	In patients at high risk of recurrence (i.e. presenting three or more risk factors), fidaxomicin should be preferred, since it was associated with a significantly lower rate of recurrence of CDI^18,34^	Agreement reached—statement to be amended The panel proposes to split this statement into two different ones (recurrent CDI versus at risk of recurrent CDI)	In patients at first CDI episode with high risk of recurrence, fidaxomicin is recommended, since it is associated with a significantly lower rate of recurrence of CDI^18,34^	Agreement reached—statement to be amended	Considering that recurrent CDI is associated with significantly higher risks of complications or death within 12 mo of the initial CDI episode,^11^ in patients with recurrent CDI fidaxomicin is recommended since it is associated with a significantly lower rate of recurrence of CDI^18,34^	Agreement reached—final statement	100%
10	In the extended-pulsed fidaxomicin regimen, the same number of fidaxomicin tablets is administered as in the standard licensed regimen, but enhanced outcomes are seen compared with vancomycin, potentially resulting in a cost-effectiveness benefit^39^	Agreement reached—statement to be amended	Extended-pulsed fidaxomicin regimen (200 mg oral tablets, twice daily on Days 1–5, then once daily on alternate days on Days 7–25) was superior to standard-dose vancomycin for sustained cure of CDI and recurrence rates were the lowest observed in a randomized clinical trial of antibiotic treatment for *C. difficile* infection without additional costs, potentially resulting in a cost-effectiveness benefit without causing dysbiosis^48^ and reducing colonization resistance	Agreement reached—statement to be amended	Extended-pulsed fidaxomicin regimen (200 mg oral tablets, twice daily on Days 1–5, then once daily on alternate days on Days 7–25) was superior to standard-dose vancomycin for sustained cure of CDI and to reduce recurrence rates without additional costs^8,49^	Agreement reached—final statement	92%
11	When available, faecal microbiota transplantation (FMT) is recommended for multiple recurrent CDI and for patients with severe complicated CDI that have deteriorated despite CDI antibiotic treatment and for whom surgery is not feasible. The risk-benefit analysis of FMT and/or surgical management should be taken on a case-by-case basis and discussed by the multidisciplinary team^18,34^	Agreement reached—statement to be amended The panel suggests splitting this sentence into two different ones	When available, faecal microbiota transplantation (FMT) is recommended for recurrent CDI and for patients with severe complicated CDI that have deteriorated despite CDI antibiotic treatment and for whom surgery is not feasible.^18,34^ FMT, in addition to standard of care antibiotics, is preferred for treatment of a second or further recurrence of CDI^18^	Agreement reached—final statement			92%
11b			The risk-benefit analysis of FMT and/or surgical management should be taken on a case-by-case basis and discussed by the multidisciplinary team^32,33^ in accordance with the centre’s availability	Agreement reached—final statement			100%
12	Bezlotoxumab should be considered for: Patients with multiple (≥3) rCDI risk factors, in addition to SoC, regardless of the severity of previous episodes	Agreement reached—final statement					92%
12b	Patients at first CDI recurrence in addition to vancomycin or fidaxomicin, when fidaxomicin was used to manage the initial CDI episode, independently of rCDI risk factors	Agreement reached—final statement					100%
12c	Patients with second or multiple CDI recurrences, in centres where FMT is not available^50^	Agreement reached—final statement					100%
12d	To balance risks/costs and benefits of its use, bezlotoxumab use should be limited in the first CDI episode only to high-risk patients and considered in patients with second or multiple CDI recurrences especially in centres where FMT is not available or contraindicated	Agreement reached—final statement					100%
13	See Table 2		Fidaxomicin is a CD narrow spectrum agent,^8^ not systemically absorbed, with limited or no activity against other enteric bacteria^51^ Resistance to fidaxomicin has rarely been reported in *C. difficile* without any effect on selection of cross-resistance with other antibiotics due to its limited activity against other enteric commensal bacteria^51^	Agreement reached—final statement			92%
14	Increased antibiotic selection pressure resulting in resistance selection (e.g. vancomycin-resistant enterococci) and side effects discourage the use of high-dose vancomycin Resistance to fidaxomicin has rarely been reported in *C. difficile* and, unlike vancomycin, there are no other treatment indications, and it is a ‘narrow spectrum’ agent with more limited activity against other enteric commensal bacteria. These pharmacological characteristics have translated into improved sustained clinical response (initial clinical response without subsequent recurrence) for patients with CDI^14^	Agreement reached—statement to be amended The panel suggests splitting this sentence into two different ones	Vancomycin increases antibiotic selection pressure resulting in resistance selection (e.g. vancomycin-resistant enterococci). Clinically, there is no benefit of a higher dose, neither in severe nor moderate CDI. Side effects such as abdominal pain and nausea discourage the use of high-dose vancomycin^18^	Agreement failed—statement to be amended and voted again	Clinically, vancomycin high-dose use (250 mg or higher 4 times a day) is discouraged due to possible side effects such as abdominal pain and nausea, whereas no benefit is observed^18^	Agreement reached—final statement	83%
15	There are clear societal benefits for treatment paradigms employing appropriate diagnosis and therapy initiation and use of targeted narrow-spectrum agents. Implementation of antimicrobial stewardship programmes in patients at risk of developing *C. difficile* infection would result in reductions in nosocomial infections caused by MDR organisms and *C. difficile*, infection-related deaths, and related therapy and management costs^41^	Agreement reached—statement to be amended	Implementation of antimicrobial stewardship and infection prevention and control programmes in persons at risk of developing bacterial infection, including CDI, would result in reductions in healthcare-associated infections caused by MDR organisms and *C. difficile*, infection-related deaths, and related therapy and management costs^52^	Agreement reached—final statement			92%
16	Cost monitoring should not be at the expense of prescriptive appropriateness, favouring fidaxomicin when appropriate. The sustainability of the use of fidaxomicin should be assessed considering the whole patient pathway related to *C. difficile* infection, including days of hospitalization, monitoring, laboratory, imaging and other outpatient activities, drugs, and not only the cost of the antibiotic therapy	Agreement failed—statement to be amended and voted again	Cost monitoring should be performed considering the global assessment patient pathway related to CDI, including days of hospitalization, monitoring, laboratory, imaging and other outpatient activities, drugs and expected compliance, besides the cost of the antibiotic therapy and promoting the choice of the right drug when appropriate	Agreement reached—statement to be amended	Cost monitoring of CDI treatment should be performed considering the global assessment of patient pathway related to CDI, including testing and other exam costs, hospital readmission rates, inpatients' and outpatients’ costs	Agreement reached—final statement	77%
17	Communication between clinicians and pharmacists remains crucial for cost monitoring. Dedicated strategies should be implemented (e.g. training in medical wards, sharing a treatment algorithm or integration tools and practical approach with the hospital’s professionals)	Agreement reached—statement to be amended	Dedicated strategies should be implemented to ensure more early and appropriate therapy for CDI, since communication between clinicians and pharmacists remains crucial for cost monitoring. Training in medical wards, and sharing a treatment algorithm or integration tools and practical approaches with the hospital’s professionals should be strongly recommended	Agreement reached—statement to be amended	Communication between clinicians and pharmacists is crucial for cost monitoring. Training in medical wards, sharing a treatment algorithm or integration tools and practical approaches with the hospital’s professionals should be strongly recommended	Agreement reached—final statement	92%
18	Considering recurrence as a pivotal factor in addressing the infection, it is imperative to consistently investigate the patient's medical history from this perspective. It is essential to appropriately consider the patient's medical history. The flow of information should facilitate retrospective tracking of the patient's history	Agreement reached—statement to be amended	Considering recurrence as a pivotal factor in addressing the infection, when feasible, the anamnesis should identify earlier CDI and the information flow should facilitate both retrospective and prospective patient’s history	Agreement reached—final statement			85%
19	The information flow should maintain the continuity of information upon hospital discharge, implementing monitoring strategies after the infection together with the HCP in charge of the patient and with the patients themselves. A follow-up call at 8 wk is suggested	Agreement failed—statement to be amended and voted again	The information flow should maintain the continuity of information upon hospital discharge. Infection details should be uniformly and clearly coded in a unique software for patient information management A follow-up call at 8 wk is suggested in order to promptly identify recurrences	Agreement reached—statement to be amended	The information flow should ensure that there is a seamless transfer of information when a patient is discharged from the hospital. Infection details should be consistently and clearly recorded using specialized software for managing patient information An 8-wk follow-up call is recommended to promptly detect any potential recurrences	Agreement reached—final statement	92%
20	Particular attention should be given to monitoring patients living in residential institutions	Agreement reached—statement to be amended.	Particular attention should be given to monitoring for CDI in high-risk subjects living in long-term care facilities/nursing homes	Agreement reached—final statement			100%
21	Patients, caregivers and/or family members should be consistently informed about the risk of recurrence and the significance of promptly reporting any recurrence to healthcare professionals	Agreement reached—statement to be amended	Patients, caregivers and/or family members should be consistently informed about the risk of transmission and recurrence and about the significance of promptly reporting any recurrence or infection in vulnerable contacts to healthcare professionals Patients at risk of low compliance should be identified and closely monitored	Agreement reached—final statement			100%

CD, *Clostridioides difficile*; CDI, *Clostridioides difficile* infection; FMT, faecal microbiota transplantation; HCP, healthcare professionals; IBD, irritable bowel disease; MPI, Multidimensional Prognostic Index; rCDI, recurrent *Clostridioides difficile* infection; SoC, standard-of-care; TCA, total cumulative agreement.

At the end of the first round of individual voting, 17 statements of 23 reached a consensus. Among them, statements 1 and 2 were agreed by the panel without suggesting any modifications, whereas the remaining 15 were suggested to be reworded or integrated. Statements indicated in Table 2 as Ex-12, Ex-13 and Ex-23, addressing management of CDI in transplanted patients and patient involvement, failed to reach an agreement and were excluded from the following steps of voting. The residual statements, addressing fidaxomicin supply, cost monitoring and post-hospitalization monitoring strategies, although they were not agreed at first instance, were revised to be submitted again to the panel for the second round of voting.

**Table 2.** Excluded statements

#	Round 1 statements	Results	Round 2 statements	Results
Ex 12	Prophylaxis of CDAD with fidaxomicin can reduce the incidence of confirmed CDAD in the HSCT population. Patients with a history of CDAD or *C. difficile* colonization prior to transplantation or at risk of recurrent CDAD after transplantation should be considered candidates for fidaxomicin prophylaxis^40^	Agreement failed—statement excluded		
Ex 13	Prophylaxis with fidaxomicin should be considered in other transplanted patients	Agreement failed—statement excluded		
Ex 16	It is suggested to always have a minimum supply of fidaxomicin available, calibrated to the different needs of hospitals, to allow for initiation of therapy when appropriate	Agreement failed—statement to be amended and voted again	In order to have equal antimicrobial stewardship programmes in different hospitals, it is desirable that, based on local and hospital epidemiology, a minimum availability of fidaxomicin is considered	Agreement failed—statement excluded
Ex 23	It is advisable to implement patient empowerment initiatives to enhance their involvement and engagement in managing the condition	Agreement failed—statement excluded		

CDAD, *Clostridioides difficile*-associated disease.

Following the suggestions received by the panel, in the second round of voting, statement 7 was integrated into statement 6, as both addressing the definition of frailty condition, whereas statement 9 was split into two different ones (8 and 9) to highlight the difference between ‘recurrent CDI’ and ‘at risk of recurrent CDI’. The ESCMID definition of ‘recurrence’ (when CDI recurs within 8 weeks after a previous episode, provided the symptoms from the previous episode resolved after completion of initial treatment)^18^ was taken as reference. Likewise, number 11, concerning faecal microbiota transplantation (FMT), and number 14, on the comparison between vancomycin and fidaxomicin, were divided into two different statements (11 was divided into 11 and 11b; 14 into 13 and 14).

The revised 20 statements, net of those that had been already finalized or excluded, were submitted to the panel for the second round of voting. At the end of this further voting step, 12 statements of 18 reached a consensus. Among them, statements 3, 5, 6, 7, 11, 11b, 15, 19, 21 and 22 were agreed by the panel without any further modifications suggested. Statements 4, 9, 10, 17, 18 and 20 reached an agreement as well, but a few rewordings and integrations were suggested. Agreement on the final wording of statement 8 was reached during the revision phase of the manuscript, after the editor’s suggestions. Once integrated or amended accordingly, these statements were presented again to the panel and were tacitly approved by each member. Conversely, statement 14, on high-dose vancomycin therapy, and statement 16, addressing availability of fidaxomicin supplies in health clinics, failed to reach a consensus but were revised to be submitted again to the panel for a final round of off-line voting. This latter confirmed disagreement about statement 16, whereas agreement was reached for statement 14.

Statement 12 was added during the revision of the manuscript following the comments of the reviewers. Agreement was reached during the first voting round after dividing the statement into four subsections.

## Discussion

Ideally, clinical recommendations should be grounded in evidence obtained from controlled clinical trials, and clinical practice should be guided by both recommendations and clinical trial findings. However, in practical terms, there may be limited research-based evidence available and the implementation of guidelines may face obstacles due to various factors, including cost-saving strategies. The present Delphi study, aligned with the Delphi modified approach,^27^ which is based on evidence-based medicine and adopts an anonymous voting process to establish opinion, was employed to achieve consensus on the most appropriate use of fidaxomicin.

This study presents results obtained by an online meeting of 13 Italian professionals with different and complementary expertise in *C. difficile* and CDI treatment (six infection disease specialists, two gastroenterologists, one internist, one microbiologist, one geriatrician, one health economist and one hospital pharmacist), two rounds of Delphi voting, and a final meeting meant to outline statements addressing the most appropriate treatment of CDI.

### Consensus statements on the use of fidaxomicin, in alignment with and supplementing the existing guidelines and on the identification of patients to be treated with fidaxomicin

Consistently, the convenience in adhering to the current guidelines on the diagnostic process^31^ and on the management of patients with CDI^32,33^ was promptly confirmed by the panel (statements 1 and 2). Moreover, statement 2 stressed the importance of pre-emptive contact isolation of patients suspected to have CDI (i.e. during sample collection).

According to the ESCMID guidelines,^18^ severe CDI is characterized by one of the following factors at presentation: fever (i.e. core body temperature >38.5°C), marked leucocytosis (i.e. leucocyte count >15 × 10^9^/L) and rise in serum creatinine (i.e. >50% above the baseline). Additional supporting factors are distension of the large intestine, pericolonic fat stranding or colonic wall thickening (including low-attenuation mural thickening) at imaging. Taking as reference this definition, the panel agreed on the identification of the risk factors for severe infection (statement 3), which take into consideration the chronological age of the patients, their clinical indicators (albuminaemia^1,34–37^ and Zar score^38,39^) and comorbidities or other conditions, namely inflammatory bowel disease (IBD), chronic kidney failure, liver failure, diabetes and cardiovascular/pulmonary diseases. The panel agreed on using the definition of severe CDI as reported in the ESCMID guidelines.

Full agreement was reached after two rounds of adjustments on these same parameters (except for the Zar score), which were marked as ‘red flags’ for risk of recurrence in patients at their first episode of CDI (statement 4). Immunocompromised patients, those recently hospitalized, and the ones under pharmacological treatment for other conditions always need to be identified. Further ‘red flags’ agreed on by the panel included: the presence or absence of an index episode and the chance to trace back a healthcare-associated origin of the infection. Other indicators with weaker but still substantial evidence for risk of recurrence were agreed to be: severity of the infection,^17^ chronic kidney failure, liver failure, diabetes, cardiovascular/pulmonary disease, parenteral nutrition,^34^ and use of concomitant antibiotics started during/after CDI diagnosis.^18,34^ Some members of the panel suggested that patient compliance (and/or caregiver accountability) can play a role in non-hospitalized patients as well.

The crucial role of IBD is highlighted by its identification as a risk factor for both severe and recurrent infections (statements 3 and 4) and by the critical role that CDI can have in these patients when causing flares (statement 5). Besides considering recent hospital admission and history of weight loss, it is important to quickly discern patients with CDI from those where diarrhoea is triggered by other causes.^53–55^

In terms of predictability of negative outcomes, frailty condition emerged as the most significant risk factor, stronger than the chronological age of the patient; the definition of ‘frailty condition’ was thoroughly discussed during the kick-off meeting and later finalized in statements 6 and 7. Since frailty level predicts mortality at 90 days more accurately than chronological age and disease severity,^44^ the need for a change of mindset in clinicians during daily management of CDIs emerged.^44,56,57^ The Multidimensional Prognostic Index (MPI)^44–46,58^ or its screening short version BRIEF-MPI,^47^ which requires a very limited amount of time, can be used as effective tools for assessing multidimensional frailty in hospitalized older patients with CDI.

In accordance with available outcomes of clinical trials,^11,12,49,59,60^ current guidelines^17,18^ and a systematic review,^61^ the consensus panel confirmed that fidaxomicin should be recommended as first-line therapy in patients at high risk of recurrence, as defined above, and in patients with rCDI, as reported in statements 8 and 9, since it is associated with a higher sustained response. Alternatively, the combination of vancomycin and FMT was demonstrated to be superior to vancomycin alone in achieving sustained resolution from CDI, as described in a recent clinical trial.^62^ As reported in statement 11, FMT in combination with standard-of-care (SoC) antibiotics was confirmed to be the preferred treatment option of second or further recurrence of CDI.^18^ FMT efficacy by itself was confirmed by the panel; this procedure is recommended for rCDI and for patients with severe CDI who have not responded to antibiotic treatment and for whom surgery is not feasible.^18,34^ Nevertheless, the main drawback of FMT lies in its current availability in a very limited number of hospitals in Italy.

Bezlotoxumab, a fully humanized monoclonal antibody directed against the binding domains of toxin B that is given as a one-time infusion in addition to an SoC antimicrobial, could be an alternative option for breaking the cycle of CDI recurrence and, based on recent evidence,^63^ should be considered for:

- patients with multiple (≥3) rCDI risk factors, in addition to SoC, regardless of the severity of previous episodes;
- patients at first CDI recurrence in addition to vancomycin or fidaxomicin, when fidaxomicin was used to manage the initial CDI episode, independently of rCDI risk factors;
- patients with second or multiple CDI recurrences, in centres where FMT is not available.^50^

This practical suggestion comes from recently published results of a real-world multicentre cohort study, including 442 patients with CDI from 2018 to 2022, collected in 18 Italian centres. This study confirmed the greater efficacy of bezlotoxumab + SoC versus SoC alone for the prevention of rCDI, already seen in previous randomized studies and a similar previous trial emulation performed using observational data^50,64,65^ Importantly, in contrast to other studies, this study was conducted in a selected population at high risk of recurrence and included the highest numbers of patients treated with fidaxomicin as SoC, compared with Spanish and US cohorts.

Although not reaching statistical significance, the benefit of bezlotoxumab + SoC on the composite outcome ((30 day recurrence and/or all-cause death) appeared to be attenuated in participants aged <70 years (*P* = 0.61) and in those who received fidaxomicin as first-line treatment (*P* = 0.71). For this reason, to balance the risks and benefits of its use, in particular weighing costs in countries with limited resources, the authors suggested limiting bezlotoxumab use in the first CDI episode only to high-risk patients (statements 12, 12b, 12c, 12d).

Agreement was obtained on the superiority of an extended-pulsed fidaxomicin regimen (200 mg oral tablets, twice daily on Days 1–5, then once daily on alternate days on Days 7–25) when compared with standard-dose vancomycin for sustained cure of CDI and to reduce rates of recurrence without additional costs^8,49^ (statement 10). Statements on fidaxomicin use for the prophylaxis of *Clostridioides difficile*-induced diarrhoea (CDAD) in persons undergoing transplantation reached a very low agreement (58% and 9%) after the first round of voting; this is likely explained by the limited literature available—only one randomized controlled trial on this topic was published—and by the high risk of side effects, including microbiome distortion, linked to its widespread use. Nevertheless, the potential benefit of a fidaxomicin-based prophylaxis in selected patients was acknowledged by some members of the panel and should be investigated further.

The panel recognized that fidaxomicin is rarely associated with resistance events thanks to its limited activity against enteric commensal bacteria. Consistently, employment of high-dose vancomycin was discouraged (statements 13/14) due to selection pressure, which results in resistant strains (e.g. vancomycin-resistant enterococci).

### Consensus statement on the obstacles involved in managing CDIs in Italy, with a specific focus on prevention and treatment of recurrent infections and the limitations affecting the use of fidaxomicin, in alignment with and supplementing the existing guidelines

Selection, prescription and administration of the most appropriate therapy are often impacted both by clinical considerations and by hospital management obligations. The panel agreed on the importance of antimicrobial stewardship (statement 15) and discussed the convenience of stocking a minimum amount of fidaxomicin in order to provide a timely treatment of CDI; however, this latter did not obtain consensus (Ex 16). Conversely, agreement (77%) was reached after online discussion and two rounds of revision on a statement addressing the importance of cost monitoring of CDI treatment (statement 16); according to the panel, this latter should be performed assessing the whole patient pathway, including testing and other exam costs, hospital readmission rates, and inpatients’ and outpatients’ costs. Notably, studies have demonstrated that initial CDI treatment with fidaxomicin results in reduced healthcare costs compared with vancomycin/metronidazole.^19–24^. Despite higher drug acquisition costs for fidaxomicin, these are offset by lower hospitalization expenses resulting from fewer recurrences, reduced complication costs and fewer GP visits compared with vancomycin. For instance, real-world studies revealed that the mean cost of a recurrent episode of CDI is in the range €9504.87 ± €8614.11,^26^ whereas in a cohort study the total cost attributable to CDI in Rome was €17 714 per patient with recurrence.^66^ It should be noted that the aforementioned health economic analyses have been supported by grants from industry. Another model set out to analyse the cost-effective sequence of antibiotic sequences as in the studies above and vancomycin/fidaxomicin was found to have a higher probability of being cost effective for an English population with characteristics of the ‘average’ CDI patient.^25^ Nevertheless, the ESCMID guideline recommendations for treating an index CDI with fidaxomicin as first-line treatment has been demonstrated by Swart *et al.*^23^ to be cost-effective compared with the NICE treatment strategy (which considers vancomycin as first-line treatment) from the UK National Health Service perspective.

Communication between clinicians and pharmacists is crucial for cost monitoring (statement 17). On a practical level, training in medical wards, and sharing treatment algorithms or integration tools and practical approaches among the hospital’s professionals was strongly recommended by the panel.

According to the preparatory analysis of this consensus, one of the issues limiting appropriate management of CDI is the complexity in recurrence identification when the patient is hospitalized in different clinics without a comprehensive medical record. The topic was discussed during the online meeting and agreement was obtained on statements 18 and 19; this highlights how technological supportive systems need to be structured and developed with the users to be effective and readily informative. It emerged also that follow-up at 8 weeks after patient dismissal should be a common practice in order to identify recurrences and implement proper treatment.

Full agreement was also reached on long-term care facilities such as nursing homes when suspecting or after identification of CDI (statement 20), and on the importance of providing patients and caregivers with consistent information about CDI and risk of recurrences when the disease is managed in an outpatient setting (statement 21). Caregivers and family members should also be informed of the risk of transmission as soon as CDI diagnosis is confirmed.

## Conclusions

Despite key advances in therapeutic options, CDI remains challenging for clinicians worldwide. The present Delphi study, aligned with the Delphi modified approach,^27^ which is centred on evidence-based medicine and adopts an anonymous voting process to establish agreement, was employed to achieve consensus on the management of CDI, on the identification of patients at risk of recurrences or severe infection, and on the most appropriate use of fidaxomicin with the aim of targeting suitability.

The enhanced benefit of this consensus document is that the results were obtained by a multidisciplinary group of 13 Italian professionals with complementary expertise in *C. difficile* management and treatment (six infection disease specialists, two gastroenterologists, one internist, one microbiologist, one geriatrist, one health economist and one hospital pharmacist). This approach was based on merging clinical experience, open panel online meeting discussions, and literature review of papers, not all of which were included in previous guidelines, due to years of publication, rigorous selection criteria and specific practical issues not addressed before.

Indeed, in practical terms, there may be limited research-based evidence available on specific issues, and the implementation of guidelines may face obstacles due to various factors, including cost-saving strategies and local or individual behaviours. Correct patient stratification will help mitigate the higher drug acquisition costs for fidaxomicin, which the panel agreed on recommending as first-line treatment for patients at risk thanks to its efficacy and narrow-spectrum antimicrobial activity.

In conclusion, this study aimed to foster clinical practice application of treatment algorithms proposed by previous guidelines, in absolute synergy. It could be an important tool to promote more appropriate and cost-effective CDI treatments in European settings with limited resources, like Italy.

## Transparency declarations

The authors declare no personal circumstances or interest that may be perceived as inappropriately influencing the representation or interpretation of reported research results. Outside the submitted work, M.B. has received funding for scientific advisory boards, travel and speaker’s honoraria from Angelini, Astellas, bioMérieux, Cidara, Gilead, Menarini, MSD, Pfizer, Shionogi, Tetraphase and Nabriva. F.G.D.R. has received a speaker’s grant from Tillotts Pharma Italy. F.C. has served as consultant to: Abbvie, MSD, Takeda, Janssen, Roche, Celgene, Bristol-Meyers Squibb, Galapagos, Gilead, Pfizer, Mundipharma, Eli-Lilly, Biogen, Ferring, Nestlè and Lionhealth; he has received lecture fees from Abbvie, Ferring, Takeda, Allergy Therapeutics, Janssen, Pfizer, Biogen, Sandoz and Tillotts Pharma, and unrestricted research grants from Giuliani, Sofar, MSD, Takeda, Abbvie, Celltrion and Pfizer. A.P. has received speaker’s honoraria from Thermo Fisher Scientific B.R.A.H.M.S. Gmb, GSK and Italfarmaco S.p.A. M.M. has received a speaker’s grant from Tillots, MSD, Angelini, Pfizer and Shionogi. M.S. has received funds for speaking at symposia organized on behalf of Gilead and Pfizer and has also received funds for research from Alifax, Hologic, Recordati and bioMérieux. M.S. is a member of the Roche advisory board for the GenMark GI panel. R.P. has received a speaker’s grant from Insmed, Gilead, SOBI and Tillotts Pharma. N.P. has received a speaker’s grant from Pfizer, Tillotts, Takeda and Qiagen. All other authors: none to declare.

## References

[1] Henrich TJ , Krakower D, Bitton A et al Clinical risk factors for severe *Clostridium difficile*-associated disease. Emerg Infect Dis 2009; 15: 415–22. 10.3201/eid1503.080312 19239754 PMC2681109

[2] Centers for Disease Control and Prevention (U.S.) . Antibiotic resistance threats in the United States, 2019. https://stacks.cdc.gov/view/cdc/82532

[3] Elliott B , Androga GO, Knight DR et al *Clostridium difficile* infection: evolution, phylogeny and molecular epidemiology. Infect Genet Evol 2017; 49: 1–11. 10.1016/j.meegid.2016.12.018 28012982

[4] ECDC . *Clostridioides difficile* infections. 2016. https://www.ecdc.europa.eu/en/clostridioides-difficile-infections

[5] ISS_EpiCentro . Sorveglianza delle infezioni da *Clostridioides difficile*. https://www.epicentro.iss.it/sorveglianza-ica/sorveglianza-infezioni-clostridioides-difficile

[6] Granata G , Petrosillo N, Adamoli L, et al Prospective study on incidence, risk factors and outcome of recurrent *Clostridioides difficile* infections. J Clin Med 2021; 10: 1127. 10.3390/jcm10051127 33800334 PMC7962640

[7] Matzaras R , Nikopoulou A, Protonotariou E et al Gut microbiota modulation and prevention of dysbiosis as an alternative approach to antimicrobial resistance: a narrative review. Yale J Biol Med 2022; 95: 479–94. 36568836 PMC9765331

[8] Alm RA , Lahiri SD. Narrow-spectrum antibacterial agents—benefits and challenges. Antibiotics (Basel) 2020; 9: 418. 10.3390/antibiotics9070418 32708925 PMC7400354

[9] Miller AC , Arakkal AT, Sewell DK et al Comparison of different antibiotics and the risk for community-associated *Clostridioides difficile* infection: a case-control study. Open Forum Infect Dis 2023; 10: ofad413. 10.1093/ofid/ofad413 37622034 PMC10444966

[10] Gonzales-Luna AJ , Carlson TJ, Garey KW. Gut microbiota changes associated with *Clostridioides difficile* infection and its various treatment strategies. Gut Microbes 2023; 15: 2223345. 10.1080/19490976.2023.2223345 37318134 PMC10274544

[11] Cornely OA , Crook DW, Esposito R et al Fidaxomicin versus vancomycin for infection with *Clostridium difficile* in Europe, Canada, and the USA: a double-blind, non-inferiority, randomised controlled trial. Lancet Infect Dis 2012; 12: 281–9. 10.1016/S1473-3099(11)70374-7 22321770

[12] Louie TJ , Miller MA, Mullane KM et al Fidaxomicin versus vancomycin for *Clostridium difficile* infection. N Engl J Med 2011; 364: 422–31. 10.1056/NEJMoa0910812 21288078

[13] Vincent C , Manges AR. Antimicrobial use, human gut microbiota and *Clostridium difficile* colonization and infection. Antibiotics (Basel) 2015; 4: 230–53. 10.3390/antibiotics4030230 27025623 PMC4790283

[14] Mullane KM , Winston DJ, Nooka A et al A randomized, placebo-controlled trial of fidaxomicin for prophylaxis of *Clostridium difficile*-associated diarrhea in adults undergoing hematopoietic stem cell transplantation. Clin Infect Dis 2019; 68: 196–203. 10.1093/cid/ciy484 29893798 PMC6321849

[15] Bishop EJ , Tiruvoipati R. Management of *Clostridioides difficile* infection in adults and challenges in clinical practice: review and comparison of current IDSA/SHEA, ESCMID and ASID guidelines. J Antimicrob Chemother 2022; 78: 21–30. 10.1093/jac/dkac404 36441203 PMC9780550

[16] Biswas JS , Patel A, Otter JA et al Reduction in *Clostridium difficile* environmental contamination by hospitalized patients treated with fidaxomicin. J Hosp Infect 2015; 90: 267–70. 10.1016/j.jhin.2015.01.015 25728208

[17] Johnson S , Lavergne V, Skinner AM et al Clinical practice guideline by the Infectious Diseases Society of America (IDSA) and Society for Healthcare Epidemiology of America (SHEA): 2021 focused update guidelines on management of *Clostridioides difficile* infection in adults. Clin Infect Dis 2021; 73: e1029–44. 10.1093/cid/ciab718 34164674

[18] van Prehn J , Reigadas E, Vogelzang EH, et al European Society of Clinical Microbiology and Infectious Diseases: 2021 update on the treatment guidance document for *Clostridioides difficile* infection in adults. Clin Microbiol Infect 2021; 27 Suppl 2: S1–21. 10.1016/j.cmi.2021.09.038 34678515

[19] Okumura H , Ueyama M, Shoji S et al Cost-effectiveness analysis of fidaxomicin for the treatment of *Clostridioides* (*Clostridium*) *difficile* infection in Japan. J Infect Chemother 2020; 26: 611–8. 10.1016/j.jiac.2020.01.018 32165072

[20] Gupta A , Ananthakrishnan AN. Economic burden and cost-effectiveness of therapies for *Clostridiodes difficile* infection: a narrative review. Therap Adv Gastroenterol 2021; 14: 17562848211018654. 10.1177/17562848211018654 PMC8170348 34104214

[21] Whitney L , Nesnas J, Planche T. Real-world budget impact of fidaxomicin versus vancomycin or metronidazole for in-hospital treatment of *Clostridioides difficile* infection. Antibiotics (Basel) 2023; 12: 106. 10.3390/antibiotics12010106 36671306 PMC9854770

[22] Medaglia AA , Mancuso A, Albano C et al *Clostridioides difficile* infection in an Italian tertiary care university hospital: a retrospective analysis. Antibiotics (Basel) 2023; 12: 837. 10.3390/antibiotics12050837. 37237740 PMC10215700

[23] Swart N , Sinha AM, Bentley A et al A cost-utility analysis of two *Clostridioides difficile* infection guideline treatment pathways. Clin Microbiol Infect 2023; 29: 1291–7. 10.1016/j.cmi.2023.06.018 37356620

[24] Chen J , Gong CL, Hitchcock MM et al Cost-effectiveness of bezlotoxumab and fidaxomicin for initial *Clostridioides difficile* infection. Clin Microbiol Infect 2021; 27: 1448–54. 10.1016/j.cmi.2021.04.004 33878506 PMC9478885

[25] Bromilow T , Holmes H, Coote L et al Cost-effectiveness analysis of antimicrobial prescribing in the treatment of *Clostridioides difficile* infection in England. Pharmacoecon Open 2023; 7: 739–50. 10.1007/s41669-023-00420-3 37306930 PMC10471526

[26] Petrosillo N , Ravasio R. Il Costo ospedaliero di trattamento di un episodio di infezione da *Clostridium difficile* in Italia. Global Reg Health Technol Assess 2017; 4: grhta.5000257. 10.5301/grhta.5000257

[27] Loblaw DA , Prestrud AA, Somerfield MR et al American Society of Clinical Oncology Clinical Practice Guidelines: formal systematic review-based consensus methodology. J Clin Oncol 2012; 30: 3136–40. 10.1200/JCO.2012.42.0489 22778311

[28] Marchesoni A , D’Angelo S, Anzidei M et al Radiologist-rheumatologist multidisciplinary approach in the management of axial spondyloarthritis: a Delphi consensus statement. Clin Exp Rheumatol 2019; 37: 575–84. 30557127

[29] Falconi M , Fazio N, Ferone D et al Use of octreotide long acting repeatable (LAR) as second-line therapy in advanced neuroendocrine tumors in different clinical settings: an Italian Delphi survey. Expert Opin Pharmacother 2020; 21: 2317–24. 10.1080/14656566.2020.1810237 32990061

[30] Fargnoli MC , Bardazzi F, Bianchi L et al Brodalumab for the treatment of moderate-to-severe psoriasis: an expert Delphi consensus statement. J Clin Med 2023; 12: 3545. 10.3390/jcm12103545 37240650 PMC10219103

[31] Crobach MJT , Planche T, Eckert C et al European Society of Clinical Microbiology and Infectious Diseases: update of the diagnostic guidance document for *Clostridium difficile* infection. Clin Microbiol Infect 2016; 22 Suppl 4: S63–81. 10.1016/j.cmi.2016.03.010 27460910

[32] Tschudin-Sutter S , Kuijper EJ, Durovic A et al Guidance document for prevention of *Clostridium difficile* infection in acute healthcare settings. Clin Microbiol Infect 2018; 24: 1051–4. 10.1016/j.cmi.2018.02.020 29505879

[33] McDonald LC , Gerding DN, Johnson S et al Clinical practice guidelines for *Clostridium difficile* infection in adults and children: 2017 update by the Infectious Diseases Society of America (IDSA) and Society for Healthcare Epidemiology of America (SHEA). Clin Infect Dis 2018; 66: e1–48. 10.1093/cid/ciy149 29462280 PMC6018983

[34] van Rossen TM , Ooijevaar RE, Vandenbroucke-Grauls CMJE et al Prognostic factors for severe and recurrent *Clostridioides difficile* infection: a systematic review. Clin Microbiol Infect 2022; 28: 321–31. 10.1016/j.cmi.2021.09.026 34655745

[35] Wiedermann CJ . Hypoalbuminemia as surrogate and culprit of infections. Int J Mol Sci 2021; 22: 4496. 10.3390/ijms22094496 33925831 PMC8123513

[36] Valiquette L , Pépin J, Do X-V et al Prediction of complicated *Clostridium difficile* infection by pleural effusion and increased wall thickness on computed tomography. Clin Infect Dis 2009; 49: 554–60. 10.1086/600879 19591596

[37] Debast SB , Bauer MP, Kuijper EJ. European Society of Clinical Microbiology and Infectious Diseases: update of the treatment guidance document for *Clostridium difficile* infection. Clin Microbiol Infect 2014; 20 Suppl 2: 1–26. 10.1111/1469-0691.12418 24118601

[38] Zar FA , Bakkanagari SR, Moorthi KMLST et al A comparison of vancomycin and metronidazole for the treatment of *Clostridium difficile*-associated diarrhea, stratified by disease severity. Clin Infect Dis 2007; 45: 302–7. 10.1086/519265 17599306

[39] Gomez-Simmonds A , Kubin CJ, Furuya EY. Comparison of 3 severity criteria for *Clostridium difficile* infection. Infect Control Hosp Epidemiol 2014; 35: 196–9. 10.1086/674851 24442086 PMC4791954

[40] Kucharzik T , Ellul P, Greuter T et al ECCO guidelines on the prevention, diagnosis, and management of infections in inflammatory bowel disease. J Crohns Colitis 2021; 15: 879–913. 10.1093/ecco-jcc/jjab052 33730753

[41] Maschmeyer G , De Greef J, Mellinghoff SC et al Infections associated with immunotherapeutic and molecular targeted agents in hematology and oncology. A position paper by the European Conference on Infections in Leukemia (ECIL). Leukemia 2019; 33: 844–62. 10.1038/s41375-019-0388-x 30700842 PMC6484704

[42] Winthrop KL , Mariette X, Silva JT et al ESCMID study Group for Infections in Compromised Hosts (ESGICH) consensus document on the safety of targeted and biological therapies: an infectious diseases perspective (soluble immune effector molecules [II]: agents targeting interleukins, immunoglobulins and complement factors). Clin Microbiol Infect 2018; 24 Suppl 2: S21–40. 10.1016/j.cmi.2018.02.002 29447987

[43] Khanna S . Management of *Clostridioides difficile* infection in patients with inflammatory bowel disease. Intest Res 2021; 19: 265–74. 10.5217/ir.2020.00045 32806873 PMC8322030

[44] Rubak T , Baunwall SMD, Gregersen M et al Frailty level at discharge predicts mortality in older patients with *Clostridioides difficile* more accurately than age or disease severity. Eur Geriatr Med 2023; 14: 583–93. 10.1007/s41999-023-00772-3 37046032 PMC10097521

[45] Dent E , Martin FC, Bergman H et al Management of frailty: opportunities, challenges, and future directions. Lancet 2019; 394: 1376–86. 10.1016/S0140-6736(19)31785-4 31609229

[46] Cruz-Jentoft AJ , Daragjati J, Fratiglioni L et al Using the multidimensional prognostic index (MPI) to improve cost-effectiveness of interventions in multimorbid frail older persons: results and final recommendations from the MPI_AGE European Project. Aging Clin Exp Res 2020; 32: 861–8. 10.1007/s40520-020-01516-0 32180170 PMC12159427

[47] Cella A , Veronese N, Custodero C et al Validation of abbreviated form of the multidimensional prognostic index (MPI): the BRIEF-MPI project. Clin Interv Aging 2022; 17: 789–96. 10.2147/CIA.S355801 35592643 PMC9112183

[48] Enoch DA , Murray-Thomas T, Adomakoh N et al Risk of complications and mortality following recurrent and non-recurrent *Clostridioides difficile* infection: a retrospective observational database study in England. J Hosp Infect 2020; 106: 793–803. 10.1016/j.jhin.2020.09.025 32987118

[49] Guery B , Menichetti F, Anttila V-J et al Extended-pulsed fidaxomicin versus vancomycin for *Clostridium difficile* infection in patients 60 years and older (EXTEND): a randomised, controlled, open-label, phase 3b/4 trial. Lancet Infect Dis 2018; 18: 296–307. 10.1016/S1473-3099(17)30751-X 29273269

[50] Meschiari M , Cozzi-Lepri A, Cervo A et al Efficacy of bezlotoxumab in preventing the recurrence of *Clostridioides difficile* infection: an Italian multicenter cohort study. Int J Infect Dis 2023; 131: 147–54. 10.1016/j.ijid.2023.04.004 37030653

[51] Cao X , Boyaci H, Chen J et al Basis of narrow-spectrum activity of fidaxomicin on *Clostridioides difficile*. Nature 2022; 604: 541–5. 10.1038/s41586-022-04545-z 35388215 PMC9635844

[52] Sadeq AA , Hasan SS, AbouKhater N et al Exploring antimicrobial stewardship influential interventions on improving antibiotic utilization in outpatient and inpatient settings: a systematic review and meta-analysis. Antibiotics (Basel) 2022; 11: 1306. 10.3390/antibiotics11101306 36289964 PMC9598859

[53] Reintam Blaser A , Deane AM, Fruhwald S. Diarrhoea in the critically ill. Curr Opin Crit Care 2015; 21: 142–53. 10.1097/MCC.0000000000000188 25692805

[54] Razik R , Rumman A, Bahreini Z et al Recurrence of *Clostridium difficile* infection in patients with inflammatory bowel disease: the RECIDIVISM study. Am J Gastroenterol 2016; 111: 1141–6. 10.1038/ajg.2016.187 27215924

[55] Polage CR , Solnick JV, Cohen SH. Nosocomial diarrhea: evaluation and treatment of causes other than *Clostridium difficile*. Clin Infect Dis 2012; 55: 982–9. 10.1093/cid/cis551 22700831 PMC3657522

[56] Pilotto A , Custodero C, Maggi S et al A multidimensional approach to frailty in older people. Ageing Res Rev 2020; 60: 101047. 10.1016/j.arr.2020.101047 32171786 PMC7461697

[57] Rao K , Micic D, Chenoweth E et al Poor functional status as a risk factor for severe *Clostridium difficile* infection in hospitalized older adults. J Am Geriatr Soc 2013; 61: 1738–42. 10.1111/jgs.12442 24083842 PMC3801297

[58] Pilotto A , Ferrucci L, Franceschi M et al Development and validation of a multidimensional prognostic index for one-year mortality from comprehensive geriatric assessment in hospitalized older patients. Rejuvenation Res 2008; 11: 151–61. 10.1089/rej.2007.0569 18173367 PMC2668166

[59] Wolf J , Kalocsai K, Fortuny C et al Safety and efficacy of fidaxomicin and vancomycin in children and adolescents with *Clostridioides* (*Clostridium*) *difficile* infection: a phase 3, multicenter, randomized, single-blind clinical trial (SUNSHINE). Clin Infect Dis 2020; 71: 2581–8. 10.1093/cid/ciz1149 31773143 PMC7744996

[60] Rao K , Zhao Q, Bell J et al An open-label, randomized trial comparing fidaxomicin to oral vancomycin for the treatment of *Clostridioides difficile* infection in hospitalized patients receiving concomitant antibiotics for concurrent infections. Clin Infect Dis 2024; 78: 277–82. 10.1093/cid/ciad606. 37797310

[61] Madoff SE , Urquiaga M, Alonso CD et al Prevention of recurrent *Clostridioides difficile* infection: a systematic review of randomized controlled trials. Anaerobe 2020; 61: 102098. 10.1016/j.anaerobe.2019.102098 31493500

[62] Baunwall SMD , Andreasen SE, Hansen MM et al Faecal microbiota transplantation for first or second *Clostridioides difficile* infection (EarlyFMT): a randomised, double-blind, placebo-controlled trial. Lancet Gastroenterol Hepatol 2022; 7: 1083–91. 10.1016/S2468-1253(22)00276-X 36152636

[63] European Medicines Agency. Zinplava (bezlotoxumab). zinplava-epar-product-information_en.pdf

[64] Hengel RL , Ritter TE, Nathan RV et al Real-world experience of bezlotoxumab for prevention of *Clostridioides difficile* infection: a retrospective multicenter cohort study. Open Forum Infect Dis 2020; 7: ofaa097. 10.1093/ofid/ofaa097 32363211 PMC7186524

[65] Johnson TM , Molina KC, Howard AH et al Real-world comparison of bezlotoxumab to standard of care therapy for prevention of recurrent clostridioides difficile infection in patients at high risk for recurrence. Clin Infect Dis 2022; 74: 1572–8. 10.1093/cid/ciab674 34665248 PMC9070853

[66] Asensio A , Di Bella S, Lo Vecchio A et al The impact of *Clostridium difficile* infection on resource use and costs in hospitals in Spain and Italy: a matched cohort study. Int J Infect Dis 2015; 36: 31–8. 10.1016/j.ijid.2015.05.013 26003403

